# Characterization of a Stably Maintained, Highly Beta-Cypermethrin-Resistant Strain of the Bird Cherry-Oat Aphid *Rhopalosiphum padi* (Hemiptera: Aphididae)

**DOI:** 10.3390/insects17101029

**Published:** 2026-10-07

**Authors:** Xinan Li, Peipan Gong, Zilan Zhao, Fengfan Wang, Qiuchi Li, Xiangrui Li, Yunhui Zhang, Xun Zhu

**Affiliations:** 1 Henan Engineering Technology Research Center for Major Invasive Alien Species Prevention and Control, School of Plant Protection and Environment, Henan Institute of Science and Technology, Xinxiang 453003, China; lixinan2019@126.com (X.L.); wangfengfanwff@163.com (F.W.); 2 State Key Laboratory for Biology of Plant Diseases and Insect Pests, Institute of Plant Protection, Chinese Academy of Agricultural Sciences, Beijing 100193, China; 19932276905@163.com (Z.Z.); liqiuchi0921@163.com (Q.L.); xrli@ippcaas.cn (X.L.); 3 The Ministry of Agriculture and Rural Affairs Key Laboratory of Integrated Pest Management of Tropical Crops, Environment and Plant Protection Institute, Chinese Academy of Tropical Agricultural Sciences, Haikou 571101, China; gpeipan@webmail.hzau.edu.cn; 4 State Key Laboratory for Quality and Safety of Agro-Products, Institute of Quality Standards and Testing Technology for Agro-Products, Chinese Academy of Agricultural Sciences, Beijing 100081, China

**Keywords:** pyrethroids, insecticide resistance, detoxifying enzymes, fitness cost, voltage-gated sodium channel

## Abstract

Insecticide resistance threatens effective pest management. In this study on *Rhopalosiphum padi*, a predominant wheat pest, a field-derived beta-cypermethrin-resistant strain (Beta-R strain) showed a 4623.50-fold resistance increase after 22 generations of beta-cypermethrin exposure and remained highly resistant (>4000-fold) after 40 generations without exposure. Beta-R strain exhibited a relative fitness of 0.92, high cross-resistance to lambda-cyhalothrin and bifenthrin, low-to-moderate resistance to sulfoxaflor, thiamethoxam, and imidacloprid, and negative or no resistance to omethoate and chlorpyrifos. The cytochrome P450 monooxygenases (P450s) and glutathione S-transferases (GSTs) contribute to this resistance. A homozygous spuer-*kdr* M918L mutation was identified in the IIS4–S6 region of the voltage-gated sodium channel gene in the Beta-R strain; however, transgenic *Drosophila melanogaster* assays showed that this mutation alone conferred only a 2.96-fold increase in beta-cypermethrin resistance. These findings show that resistant of *R. padi* to beta-cypermethrin can be retain high-level resistance without insecticide exposure, while also conferring high-level cross-resistance to other pyrethroids and low or negative resistance to other insecticides with different modes of action, with the resistance phenotype linked to upregulated P450/GST activities and an M918L mutation that makes only a minor contribution. These findings have significant implications for the management of resistance to pyrethroid insecticides in *R. padi*.

## Simple Summary

Wheat is one of the main food crops in the world, but its production is harmed by aphid species, involving the bird cherry-oat aphid *Rhopalosiphum padi*. The management of agricultural pests mainly depends on the widespread use of chemical insecticides. Pyrethroids, which are one of the major classes of insecticides that are frequently used for controlling aphids. However, the long-term and widespread use of pyrethroid insecticides has led to the development of insecticide resistance in many pests. Our findings revealed the characterization of a stably maintained, highly beta-cypermethrin-resistant strain in *R. padi*. The research results are of great significance for facilitating the development of effective methods for managing *R. padi* and the rational use of insecticides.

## 1. Introduction

The bird cherry-oat aphid, *Rhopalosiphum padi* (Linnaeus), is a major wheat pest worldwide. It attacks all major cereals and pasture grasses but especially barley, oat, maize, and wheat, as well as other Gramineae species; it causes significant damage by direct feeding and through the transmission of plant viruses [1]. The application of chemical insecticides is the most effective strategy for controlling pests. However, *R. padi* field populations are becoming increasingly resistant to some insecticides, including organophosphates, carbamates, and pyrethroids [2,3,4].

Pyrethroids are a major class of insecticides frequently used for controlling pests, including aphids [5]. Because of their high efficiency, low toxicity, and the minimal residues left behind, which ensures crops are safe to consume after their use, pyrethroids may be further developed and improved for future field applications. However, the long-term widespread application of pyrethroids has led to the development of resistance in many pests, including *Aphis gossypii* [5] and *Myzus persicae* [6]. As an increasing number of pests become resistant to pyrethroids, their value and utility are likely to decrease.

The inheritance of insecticide resistance limits the effective chemical control of pests and increases the rate of resistance formation [7]. The genetic basis for pyrethroid resistance has been reported in several insects, including *Musca domestica* and *Plutella xylostella* [8,9]. Insecticide resistance of insect populations is usually accompanied by fitness costs (e.g., low survival rate, low fecundity, and reduced longevity), which may affect the rate of resistance spread [10,11]. The fitness costs associated with pyrethroid insecticide resistance have been reported in *R. padi* [12]. Previous research has also indicated that insecticide resistance with fitness costs may be minimized by stopping the use of the insecticide for a certain period [13,14]. However, insecticide resistance without fitness costs may contribute to the rapid development and spread of resistance in populations [15].

Clarifying how pests respond to insecticides in terms of biological and biochemical activities may be relevant for characterizing insecticide resistance mechanisms and for improving pest management programs. The long-term use of insecticides often leads to insect populations developing resistance to additional insecticides. Elucidating the spectrum of cross-resistance or even multiple resistance is essential for prolonging the utility of insecticides in the field. High cross-resistance between different pyrethroids has been confirmed in *R. padi* [16,17]. Additionally, a thiamethoxam-resistant strain of the cotton aphid *A. gossypii* exhibiting cross-resistance to bifenthrin and alpha-cypermethrin has been identified [18].

Changes in insect metabolism or behavior and target-site mutations are common in insecticide-resistant insects [19]. Insecticide resistance mediated by cytochrome P450 monooxygenase (P450), carboxylesterase (CarE), and glutathione S-transferase (GST) has been detected in many species, including *M. persicae* [20], *Drosophila melanogaster* [21], and *Schizaphis graminum* [22]. Multiple mutation sites associated with pyrethroid resistance have been identified in voltage-gated sodium channel (*VGSC*)-encoding genes in different insect species [17,23,24]. A previous study demonstrated that the resistance of *Tuta absoluta* to pyrethroid insecticides is due to increased insecticide detoxification and mutations in *VGSC* [25]. A recent study showed that the heterozygous super-*kdr* mutation M918L and multiple cytochrome P450s are associated with bifenthrin and lambda-cyhalothrin resistance of *R. padi* [16,17].

The aim of this study was to characterize beta-cypermethrin resistance in *R. padi* and to investigate the potential mechanisms by which this resistance can reach and persist at a high level. The specific objectives were: (1) to quantify the resistance level and its stability across generations; (2) to evaluate the associated fitness cost using the age-stage, two-sex life-table approach; (3) to determine cross-resistance to other insecticides; (4) to identify the roles of detoxifying enzymes and (5) to identify VGSC target-site mutations and functionally confirm the M918L mutation using transgenic *D. melanogaster*. These findings will facilitate effective resistance management and the rational use of pyrethroids against *R. padi.*

## 2. Materials and Methods

### 2.1. Insects, Insecticides, Chemicals, and Synergists

The *R. padi* aphids used in this study were originally collected in 2018 from a field population with high beta-cypermethrin resistance (130-fold greater than a sensitive, indoor strain) in Shizuishan, Ningxia (NXS) (39°5′57″ N, 106°44′51″ E), China. Isofemale lines were established from the NXS field population. The adults from each isofemale line were bioassayed using the LC_50_ concentration of beta-cypermethrin with a slightly modified leaf dip bioassay, as detailed below. Lines showing the lowest adult mortality were retained as the resistant clone (Beta-R), and the line showing the highest mortality was retained as the susceptible clone (SS). The Beta-R clone was subsequently propagated with intermittent beta-cypermethrin exposure every two or three generations. All the aphids were reared on Lunxuan 987 wheat seedlings in a climate-controlled chamber maintained at 20 °C ± 1 °C with 60% ± 10% relative humidity and a 16-h light:8-h dark photoperiod. The wild-type (A-Dmel) and M918 mutant (T-Dmel) *Drosophila melanogaster* were provided by Fungene Biotechnology (Beijing, China). Details about insecticides, chemicals, and synergists are given in Text S1 of the Supplementary Materials.

### 2.2. Insecticide Bioassays

A slightly modified leaf dip method [26] was used to determine the susceptibility of *R. padi* to insecticides. Briefly, insecticides were dissolved in N, N-dimethylformamide (DMF) to produce stock solutions, and omethoate EC was used directly as a stock solution. The stock solutions were then diluted with water containing 0.1% (*v*/*v*) polyoxyethylenesorbitan monooleate (Tween 80) to generate a gradient series comprising five concentrations. At least 30 aphids were treated with each insecticide concentration, with three replicates per treatment. The leaves with adult aphids (no alatae) were immersed in the insecticide solutions for 3–5 s, after which they were placed on damp filter paper in a disposable culture dish. The controls were leaves harboring wheat aphids immersed in distilled water containing 0.1% (*v*/*v*) Tween-80 and DMF. Mortality was assessed after 24 h using a stereo microscope; the mortality of the control aphids was less than 10%. The aphids were considered dead if they did not move when prodded with an anatomical needle. Corrected mortality was calculated using Abbott’s formula. The median lethal concentration (LC_50_), 95% confidence interval (95% CI), and slope ± standard errors (SE) of the probit regression line—the latter reflecting the heterogeneity of the test population’s response to the insecticide—were estimated by probit analysis using Data Processing System (DPS software, version 7.05) [27]. *p* < 0.05 indicates a significant linear relationship between the logarithm of insecticide dose and the probability of death. The relative resistance ratio (RLR) was estimated using the following equation: RLR = LC_50_ of the Beta-R strain/LC_50_ of the SS strain.

### 2.3. Life-Table Analysis

The establishment of the life table and the analysis were performed according to the age-stage, two-sex life-table theory using the TWOSEX-MSChart program (version 2025.03.23) [28,29]. For each strain, apterous adults that had not reproduced were randomly selected for the life-table study (details in Supplementary Materials Text S2). The raw life-table data for the *R. padi* Beta-R and SS strains were analyzed according to the age-stage, two-sex life-table theory using the TWOSEX-MSChart program (version 2025.03.23) [30,31]. The variance and standard error of life-table parameters were calculated using the bootstrap procedure included in TWOSEX-MSChart (version 2025.03.23), with 100,000 random resamplings [30,31]. The differences in life-table parameters between the Beta-R and SS strains were assessed using the paired-bootstrap test based on the CIs of the differences at the 5% significance level implemented in TWOSEX-MSChart (version 2025.03.23) [32,33]. The net reproductive rate (*R*_0_) was calculated as *R*_0_ = ∑*l_x_m_x_* where *x* is the age in days, *l*_x_ is the proportion of individuals surviving to age *x*, and *m_x_* is the number of offspring produced per aphid at age *x*. The intrinsic rate of increase (*r*) was estimated using the Euler-Lotka equation $\sum_{i=1}^{N}e^{-r(x+1)}$*l_x_m_x_* = 1 with age indexed from 0. The mean generation (*T*) time was then calculated as *T* = ln*R*_0_/*r*. The relative fitness (*r_f_*) of the Beta-R strain was calculated as *r_f_* = *r* of the Beta-R strain/*r* of the SS strain [12].

### 2.4. Synergist Bioassays

Insecticide toxicities in the presence and absence of the synergists piperonyl butoxide (PBO), triphenyl phosphate (TPP), and diethyl maleate (DEM) were determined using the bioassay method described above. The maximum sublethal doses for PBO, TPP, and DEM for the SS strain were also determined using the same bioassay method. The maximum dose that resulted in a mortality of 0% was adopted as the maximum sublethal concentration. In accordance with a published method [34], aphids were treated with 625 mg/L TPP, PBO, or DEM 2 h before insecticide treatments. The synergistic ratio (SR) was calculated as LC_50_ (insecticide alone)/LC_50_ (insecticide + synergist), and the relative synergism ratio (RSR) was calculated as the SR of the Beta-R strain with the synergist divided by the SR of the SS strain with the synergist, using DPS software (version 7.05). The inclusion of 1 in the 95% CI of the SR indicated that the synergism was not significant.

### 2.5. Enzyme Activity Analysis

Twenty milligram batches of apterous adults (five biological replicates per strain) were manually homogenized in 180 μL of ice-cold 0.1 M sodium phosphate buffer (pH 7.6) containing 1 mM ethylenediaminetetraacetic acid (EDTA), 1 mM dithiothreitol (DTT), 1 mM 1-phenyl-2-thiourea (PTU), 1 mM phenylmethylsulfonyl fluoride (PMSF), and 20% glycerol. Homogenates were then centrifuged at 12,000× *g* for 15 min at 4 °C in the Fresco^TM^ 21 microcentrifuge (Thermo Fisher Scientific, Waltham, MA, USA). The supernatants (i.e., enzyme solutions) were collected and analyzed for CarE, GST, and P450 activities as well as their protein contents (see Text S3 in the Supplemental Materials). Briefly, an insect mixed-function oxidase ELISA kit (Huabaitai Biotechnology Corporation, Beijing, China) was used to assay P450 levels as previously described [35]. CarE and GST activities were determined using α-naphthyl acetate (α-NA) and 1-chloro-2,4-dinitrobenzene (CDNB) as substrates, respectively, as previously described [36,37]. The protein content of the enzyme solution was determined using the Bradford method with bovine serum albumin (BSA) as the standard [38]. The significance of the differences in enzyme activities between the SS and Beta-R strains was determined by Student’s *t* test using GraphPad Prism 8.0.1 (GraphPad Software Inc., La Jolla, CA, USA), with the threshold of significance set at *p* < 0.05. All data are presented as means ± SE of five replicates.

### 2.6. Detection of Mutations in the R. padi VGSC Gene

Total RNA was extracted from 10 apterous adult aphids using the RNAprep Pure Tissue Kit (Tiangen, Beijing, China), with five replicates per strain. Full-length *RpVGSC* sequences (*RpVGSC*-H1 and *RpVGSC*-H2) were amplified using gene-specific primer pairs (Table S1) and LA Taq Polymerase (Takara, Dalian, China) as previously described [12]. The PCR products were analyzed in agarose gels, extracted, cloned into plasmids, and purified (see Text S4 in the Supplemental Materials). Ten positive clones per sample were randomly selected for bidirectional sequencing by Tsingke Biotech (Beijing, China). The sequencing results were used to predict the encoded amino acid sequences, which were then used to screen the NCBI database. Additionally, chromatographic peaks were checked using Chromas software (version 2.6.4). Details are given in Text S4 in the Supplementary Materials.

## 3. Results

### 3.1. High-Level Resistance to Beta-Cypermethrin in R. padi

**Figure 1:**
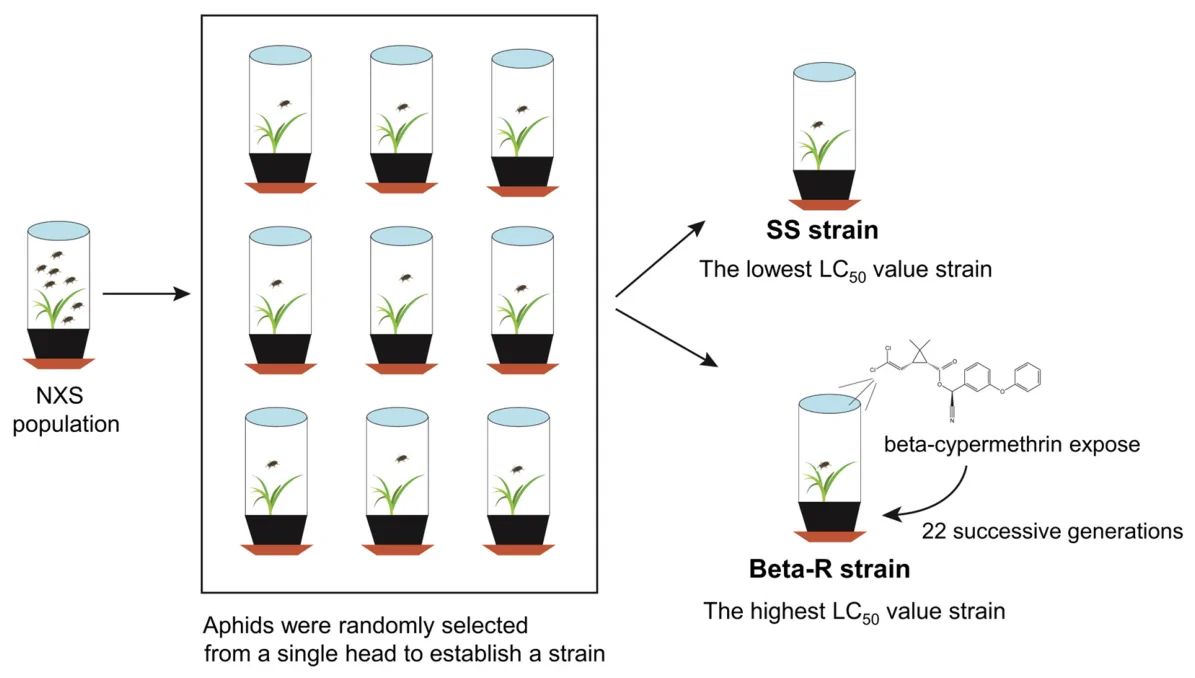
Information regarding the genetic background of the strains used in this study.

The resistant and susceptible strains were obtained from multiple isofemale lines in the NXS population (Figure 1). The Beta-R strain was already 106.77-fold resistant at generation 0. After 22 generations of intermittent beta-cypermethrin exposure, its LC_50_ had increased further, reaching a 4623.50-fold resistance ratio relative to the SS strain (Table 1 and Table S2). After 40 generations without insecticide exposure, the Beta-R strain remained highly resistant to beta-cypermethrin (4160.44-fold) at generation 62 (Table 1).

### 3.2. Relative Fitness Cost

Life-table parameters for the SS and Beta-R strains after 62 generations are presented in Table 2 (raw data in Supplementary Materials Texts S5 and S6). Compared with the SS strain, the first instar nymph, third instar nymph, and pre-adult developmental periods, total longevity, and total pre-reproductive period (TPRP) for the Beta-R strain were significantly prolonged (*p* < 0.05). However, there were no significant differences between the Beta-R and SS strains in terms of the second instar nymph and fourth instar nymph developmental periods, adult longevity, adult pre-reproductive periods (APRP), reproductive periods, and fecundity (*p* > 0.05). The population life-table parameters for the SS and Beta-R strains are listed in Table 2. There was no significant difference in the net reproductive rate (*R*_0_) of the Beta-R and SS strains (*p* = 0.9745). However, compared with the SS strain, the intrinsic rate of increase (*r*) was lower for the Beta-R strain (*p* < 0.0001), and mean generation time (*T*) was significantly longer for the Beta-R strain than for the SS strain (*p* < 0.0001). The calculated *r_f_* (i.e., *r* of the Beta-R strain/*r* of the SS strain = 0.3594/0.3884 = 0.92) indicated that the insecticide resistance of the Beta-R strain had a relative fitness cost.

### 3.3. Resistance of the Beta-R Strain to Other Insecticides

The resistance of the Beta-R and SS strains to insecticides was determined after 62 generations (Table 3 and Table S3). The results indicated that the Beta-R strain showed high resistance to lambda-cyhalothrin (899.68-fold) and bifenthrin (298.64-fold), moderate resistance to sulfoxaflor (20.79-fold) and thiamethoxam (12.21-fold), and minimal resistance to imidacloprid (6.28-fold) and abamectin (6.75-fold). In addition, negative resistance was detected for omethoate (0.54-fold), while no significant difference in resistance was observed for chlorpyrifos (0.67-fold).

### 3.4. Synergists and Enzyme Activities

**Figure 2:**
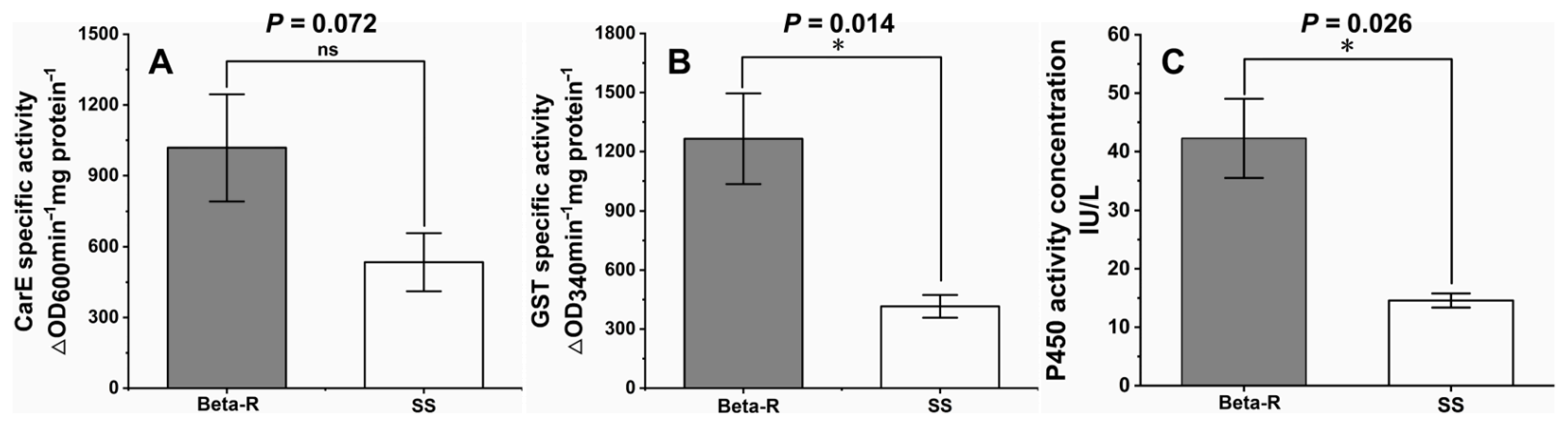
Specific activities of carboxylesterase (CarE, (**A**)), glutathione-S-transferases (GSTs, (**B**)), and cytochrome P450 monooxygenases (P450s, (**C**)) in *Rhopalosiphum padi* beta-cypermethrin-susceptible (SS) and beta-cypermethrin-resistant (Beta-R) strains after their 62nd generations. Data are presented as means ± SE (*n* = 5). * Significant difference according to Student’s *t* test (*p* < 0.05). ns indicates no significant difference.

The analyses of enzyme activities indicated that P450 and GST activities were respectively 2.35-fold (*F* = 11.978, *p* = 0.026) and 2.90-fold (*F* = 17.166, *p* = 0.014) higher in the Beta-R strain than in the SS strain (Figure 2). We failed to detect statistically significant differences in CarE activity between the two strains (*F* = 5.927, *p* = 0.072) (Figure 2). Additionally, the effects of the synergists PBO, TPP, and DEM on beta-cypermethrin susceptibility in the Beta-R and SS strains after their 62nd generations are presented in Table 4, Tables S4 and S5. The resistance of the Beta-R strain was suppressed by the oxidase inhibitor PBO (7.16-fold) and the glutathione depleter DEM (3.33-fold), whereas the effect of the esterase inhibitor TPP was not significant. The relative synergistic effects of PBO and DEM on the Beta-R strain were 2.82-fold and 1.67-fold, respectively (Table 4).

### 3.5. Detection of Mutations of the VGSC Gene and Transgenic D. melanogaster

**Figure 3:**
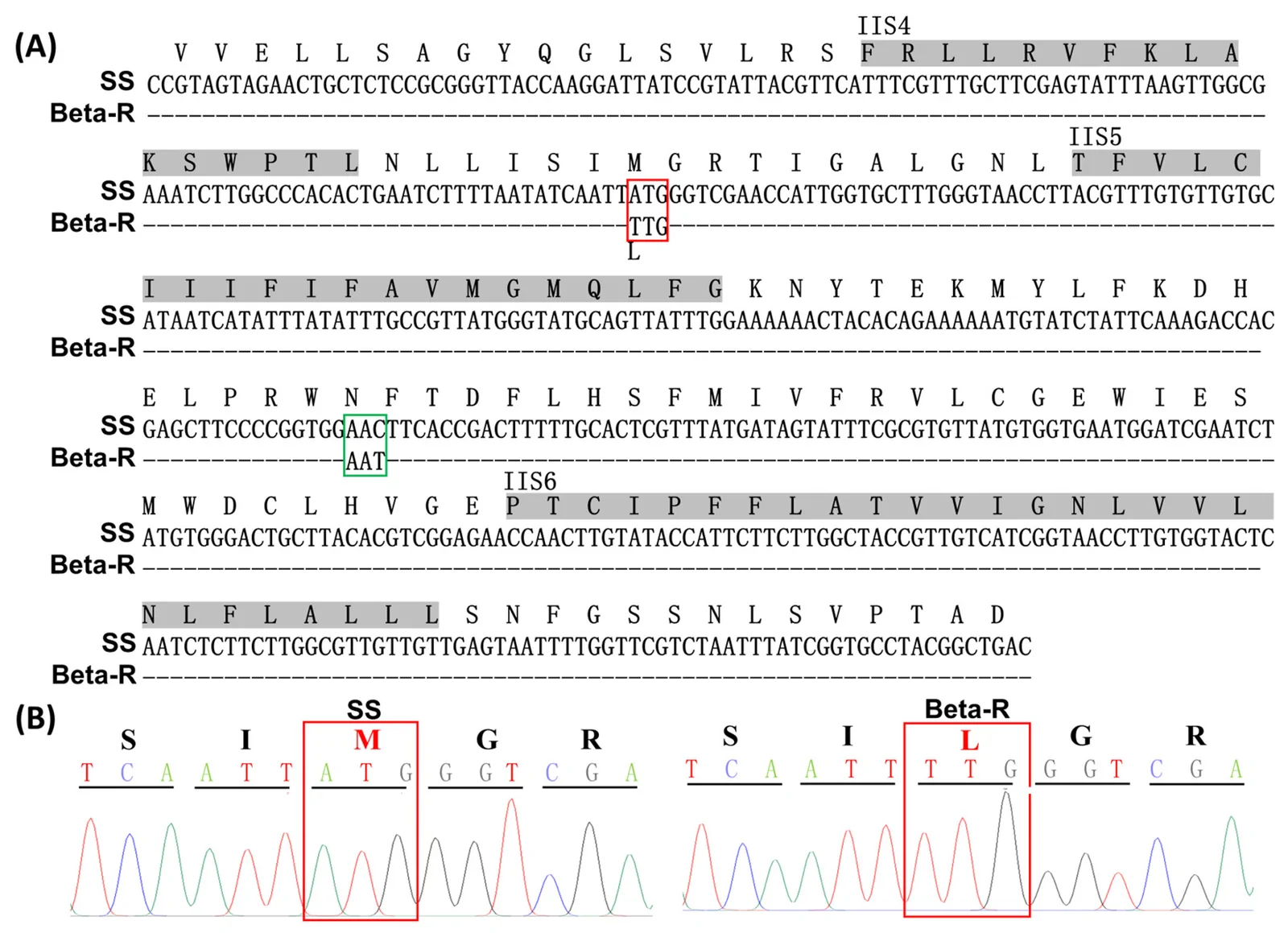
Sequence alignment (**A**) and chromatograms (**B**) of the target-site mutations in the voltage-gated sodium channel gene IIS4–S6 region in *Rhopalosiphum padi* beta-cypermethrin-susceptible (SS) and beta-cypermethrin-resistant (Beta-R) strains after their 62nd generations. M, methionine; L, leucine.

The *RpVGSC*-H1 and *RpVGSC*-H2 sequences of the Beta-R and SS strains were amplified by PCR, visualized by 1.0% agarose gel electrophoresis (Figure S1), and cloned, resulting in fragments of approximately 3400 bp and 2800 bp, respectively. The encoded amino acid sequences were determined and compared with known sequences. The *RpVGSC*-H1 and *RpVGSC*-H2 amino acid sequences of the SS strain were 100% similar to the QTH36189.1 and QTH36190.1 sequences in the NCBI database, respectively (Figure S2). The ATG-to-TTG mutation in the Beta-R strain sequence resulted in a methionine-to-leucine amino acid change, which was confirmed to be the super-*kdr* M918L mutation (Figure 3A). In contrast, the AAC-to-AAT mutation did not alter the amino acid sequence (Figure 3A). No other mutations were detected in the *VGSC* gene sequences of the Beta-R strain (Figure S2). Moreover, the ATG-to-TTG mutations in all Beta-R strain samples were detected as single peaks in the sequence chromatograms (Figure 3B), indicating that the super-*kdr* M918L mutation in the IIS4–S6 region of the *VGSC* gene was homozygous. We successfully obtained a mutant strain of transgenic *D. melanogaster* containing homozygous M918L (Figure S3). Toxicity tests of beta-cypermethrin against wild-type (A-Dmel) and M918L mutant (T-Dmel) *D. melanogaster* showed that the resistance level ratio of T-Dmel to beta-cypermethrin was significantly 297-fold higher than that of A-Dmel (Table 5 and Table S6).

## 4. Discussion

Effective pest management relies primarily on the application of insecticides. Since the 1970s, pyrethroids have been one of the most widely used insecticides worldwide for the control of agricultural pests. However, many pest species have developed resistance to pyrethroids, including *A. gossypii* [5] and *M. persicae* [6]. Additionally, several recent studies have revealed that some *R. padi* field populations are highly resistant to beta-cypermethrin, lambda-cyhalothrin, deltamethrin, and bifenthrin [4,16]. In the current study, beta-cypermethrin resistance developed *R. padi* that was highly resistant to beta-cypermethrin after exposure. Similar results were found in resistance of *Bemisia tabaci* to afidopyropen [39], and resistance of *R. padi* to lambda-cyhalothrin [17]. Additionally, the high beta-cypermethrin resistance of *R. padi* can be stably inherited in our study. In a previous study, in silico analysis of field data from Brazil showed that the reversal of pyrethroid resistance in *Aedes aegypti* could take decades [40].

The development of insecticide resistance is often accompanied by fitness costs. The fitness costs related to the imidacloprid resistance of *R. padi* strains include significantly prolonged nymph developmental stages, shortened life spans, and decreased fecundity and life-table parameters (e.g., *r*, *λ*, *R*_0_, and *T*) [41]. Previous research has indicated that the bifenthrin resistance of *R. padi* (1033.04-fold) showed a relative fitness of 0.69 [17]; however, the lambda-cyhalothrin resistance of *R. padi* (194.70-fold) showed a relative fitness of 0.94 [12]. In the current study, fecundity and *R*_0_ of Beta-R strain did not differ significantly from those of the SS strain. However, the high beta-cypermethrin resistance of the Beta-R strain was accompanied by fitness costs, including a prolonged *T* and a decreased r, resulting in a relative fitness of 0.92. Higher beta-cypermethrin resistance may persist in *R. padi* populations because of its hereditary stability. This high resistance could pose a major challenge to the effective use of pyrethroids in controlling wheat aphids. Nevertheless, the existence of fitness costs is beneficial in resistance management.

Long-term use of insecticides often leads to insect populations also developing resistance to other insecticides. For example, in an earlier study, an *R. padi* strain resistant to bifenthrin (1033.04-fold) exhibited cross-resistance to deltamethrin (974.48-fold) [17]. In our study, the highly beta-cypermethrin-resistant Beta-R strain was also highly resistant to lambda-cyhalothrin (899.68-fold) and bifenthrin (298.64-fold). It is conceivable that a similar mechanism mediates resistance to various pyrethroid insecticides [42]. Additionally, the Beta-R strain was moderately resistant to sulfoxaflor and thiamethoxam, low resistant to imidacloprid and abamectin, negatively resistant to omethoate, and no resistance to chlorpyrifos. This is consistent with the results of an earlier investigation on negative cross-resistance between pyrethroid insecticides and organophosphates [43]. These findings have important implications for the use of pyrethroids to control insect pests and highlight the need for rational pesticide use.

Several reports describe the P450-mediated resistance of many pests, e.g., increases in the catalytic activity of P450s contribute to the pyrethroid resistance of *R. padi* [16,17] and *A. gossypii* [5], whereas GSTs help detoxify cellular toxins and contribute to metabolism-based resistance to pyrethroids [44,45]. In the current study, the P450 and GST activities of the Beta-R strain were respectively 2.35-fold and 2.90-fold higher than those of the SS strain, and the relative synergistic effects of PBO and DEM on the Beta-R strain were 2.82-fold and 1.67-fold, respectively. These results suggest that P450s and GSTs contribute to the high beta-cypermethrin resistance.

Target-site mutations are common in insecticide-resistant insects. Multiple mutations or co-mutation sites associated with pyrethroid resistance have been identified in *VGSC* genes in diverse insect species [23,24,46]. For example, the M918L and L925I co-mutations in greenhouse whitefly have led to a 662-fold increase in bifenthrin resistance, whereas single T929I or L925I mutations increased insecticide resistance by 263-fold and 471-fold, respectively [47]. Furthermore, the resistance of *R. padi* to lambda-cyhalothrin (158.76-fold) and bifenthrin (1033.04-fold) is reportedly associated with a single heterozygous mutation (i.e., super-*kdr* M918L) [16,17]. Our study is the first to detect a single homozygous super-*kdr* M918L mutation in *R. padi* with high beta-cypermethrin resistance. Similar results have been observed in *A. gossypii* that are highly resistant to pyrethroids [5]. However, transgenic *D. melanogaster* assays showed that this mutation alone conferred only a 2.96-fold increase in beta-cypermethrin resistance, indicating that the M918L mutation makes only a minor contribution.

## 5. Conclusions

In conclusion, the current study revealed that *R. padi* can retain high-level resistance to beta-cypermethrin without insecticide exposure, while also exhibiting high-level cross-resistance to other pyrethroids and low or negative cross-resistance to insecticides with different modes of action. This resistance phenotype was associated with upregulated P450/GST activities and an M918L mutation that makes only a minor contribution. Given that the mechanisms underlying pest resistance to insecticides are complex, future research should focus on the molecular level by investigating the transcriptional expression levels of all detoxification enzyme genes and comprehensively analyzing the causes of this resistance. High-level resistance to pyrethroid insecticides has restricted the use of these pesticides. To enhance the utility of pyrethroids, rotating the application of insecticides with different insecticidal mechanisms based on the results of resistance monitoring may be an effective strategy for preventing or delaying the development of wheat aphids resistant to pyrethroids.

## Figures and Tables

**Table 1:** Levels of resistance to beta-cypermethrin of *Rhopalosiphum padi* beta-cypermethrin-susceptible (SS) and beta-cypermethrin-resistant (Beta-R) strains.

Strain	Generation	N	Slope ± SE	LC_50_ (mg/L) (95% CI)	*p*-Values	RLR
SS		659	0.85 ± 0.05	3.16 (2.14–4.65)	0.0006	
Beta-R	G_0_	752	1.11 ± 0.09	337.38 (230.73–493.34)	0.0012	106.77
	G_4_	614	0.84 ± 0.13	886.42 (336.94–2141.33)	0.0072	280.51
	G_7_	650	2.24 ± 0.17	1257.31 (1046.34–1510.82)	0.0010	397.88
	G_10_	436	1.12 ± 0.10	4669.85 (2807.42–7767.83)	0.0079	1477.80
	G_22_	635	1.40 ± 0.10	14,610.27 (11,222.21–19,021.21)	0.0055	4623.50
	G_62_	554	1.27 ± 0.12	13,146.98 (9406.70–18,374.45)	0.0097	4160.44

**N**, Number of aphids tested; 95% CI, 95% confidence interval; ***p*-Values**, *p* < 0.05 indicates a significant linear relationship between the logarithm of insecticide dose and the probability of death. RLR, relative resistance ratio, RLR = LC_50_ of the Beta-R strain/LC_50_ of the SS strain. Generations 0 to 22 of the Beta-R strain were continuously exposed to beta-cypermethrin. Generations 22 to 62 of the Beta-R strain were not exposed to any insecticides.

**Table 2:** Developmental durations and population life-table parameters for *Rhopalosiphum padi* beta-cypermethrin-susceptible (SS) and beta-cypermethrin-resistant (Beta-R) strains after their 62nd generations.

Index	SS Strain		Beta-R Strain		Statistical Analysis	
	*N*	Mean ± SE	*N*	Mean ± SE	95% CI	*p*-Value
First instar (d)	104	1.47 ± 0.05	104	2.00 ± 0.01	(0.43, 0.63)	<0.0001
Second instar (d)	103	1.90 ± 0.04	104	1.82 ± 0.04	(−0.02, 0.20)	0.1455
Third instar (d)	101	1.80 ± 0.04	103	1.97 ± 0.02	(0.08, 0.25)	0.0001
Fourth instar (d)	100	2.01 ± 0.02	99	2.01 ± 0.02	(−0.05, 0.05)	1.000
Pre-adult (d)	100	7.16 ± 0.066	99	7.80 ± 0.05	(0.48, 0.80)	<0.0001
Adult longevity (d)	100	14.83 ± 0.61	99	16.30 ± 0.60	(−0.19, 3.13)	0.0853
Total longevity (d)	104	21.34 ± 0.67	104	23.28 ± 0.68	(0.07, 3.81)	0.0422
APRP (d)	100	0.00 ± 0.00	99	0.02 ± 0.02	(−0.02, 0.06)	0.5120
TPRP (d)	100	7.16 ± 0.07	99	7.82 ± 0.06	(0.49, 0.83)	<0.0001
Reproductive period (d)	100	10.36 ± 0.26	99	10.42 ± 0.22	(−0.60, 0.73)	0.8501
Fecundity (offspring/female)	100	72.71 ± 1.63	99	73.56 ± 1.45	(−3.40, 5.07)	0.6969
Intrinsic rate of increase/*r*	104	0.3884 ± 0.0046	104	0.3594 ± 0.0033	(0.0180, 0.0404)	<0.0001
Net reproductive rate/*R*_0_	104	69.92 ± 2.06	104	70.02 ± 2.06	(−5.64, 5.83)	0.9745
Mean generation time/*T*	104	10.93 ± 0.11	104	11.8 2± 0.08	(0.61, 1.16)	<0.0001
^c^ *r_f_*				0.92		

*N*, Number of aphids tested; APRP, adult pre-reproductive period; TPRP, total pre-reproductive period; standard errors (SE) were estimated by bootstrapping (100,000 random re-samplings); the difference between two strains was evaluated by conducting a paired bootstrap test; if the confidence interval included 0, there was no difference at the 5% level; ^c^ *r_f_* = *r* of the Beta-R strain/*r* of the SS strain.

**Table 3:** Multiple resistance spectrum of *Rhopalosiphum padi* beta-cypermethrin-susceptible (SS) and beta-cypermethrin-resistant (Beta-R) strains after their 62nd generation.

Insecticide	Strain	*N*	Slope ± SE	LC_50_ (mg/L) (95% CI)	*p*-Values	RLR (95% CI)
Lambda- cyhalothrin	SS	889	0.89 ± 0.05	5.09 (3.34–7.75)	0.0025	
	Beta-R	798	0.64 ± 0.07	4582.51 (1833.24–11,454.84)	0.0004	899.68 (328.31–2465.42) *
Bifenthrin	SS	921	0.91 ± 0.08	5.54 (3.51–8.72)	0.0013	
	Beta-R	800	0.79 ± 0.05	1653.22 (914.38–2989.08)	0.0008	298.64 (141.51–630.24) *
Sulfoxaflor	SS	997	0.96 ± 0.07	6.80 (4.57–10.12)	0.0010	
	Beta-R	958	0.93 ± 0.07	141.53 (97.81–204.78)	0.0009	20.79 (12.08–35.77) *
Thiamethoxam	SS	993	0.74 ± 0.05	4.74 (3.25–6.94)	0.0007	
	Beta-R	836	0.60 ± 0.03	57.98 (45.00–74.71)	0.0004	12.21 (7.74–19.28) *
Imidacloprid	SS	898	0.70 ± 0.06	10.75 (7.23–15.97)	0.0012	
	Beta-R	577	0.88 ± 0.04	67.49 (55.79–81.64)	0.0002	6.28 (4.04–9.75) *
Abamectin	SS	832	1.69 ± 0.13	7.64 (5.78–10.10)	0.0059	
	Beta-R	746	0.97 ± 0.05	51.58 (41.86–63.57)	0.0029	6.75 (4.76–9.56) *
Omethoate	SS	627	1.12 ± 0.09	115.02 (78.62–168.28)	0.0011	
	Beta-R	1043	1.32 ± 0.09	62.51 (45.58–85.72)	0.0008	0.54 (0.33–0.89) *
Chlorpyrifos	SS	939	3.38 ± 0.68	2.58 (1.74–3.82)	0.0153	
	Beta-R	677	2.43 ± 0.40	1.74 (0.75–4.02)	0.0263	0.67 (0.27–1.70)

***N***, Number of aphids tested; 95% CI, 95% confidence interval; ***p*-Values**, *p* < 0.05 indicates a significant linear relationship between the logarithm of insecticide dose and the probability of death. Relative resistance ratio (RLR) = LC_50_ of the Beta-R strain/LC_50_ of the SS strain; *, the non-inclusion of 1 in the 95% CI indicates a significant difference in LC_50_ values of the insecticides between the two strains.

**Table 4:** Synergistic effects of TPP, DEM, and PBO in the *Rhopalosiphum padi* beta-cypermethrin-susceptible (SS) and beta-cypermethrin-resistant (Beta-R) strains after their 62nd generation.

Strain	Beta + Synergist	*N*	Slope ± SE	*p* Values	LC_50_ (mg/L) (95% CI)	SR (95% CI)	RSR
SS	Beta	659	0.85 ± 0.05	0.0006	3.16 (2.14–4.65)	-	-
	Beta + PBO	654	0.93 ± 0.04	0.0033	1.25 (0.90–1.72)	2.54 (1.53–4.19) *	
	Beta + DEM	776	0.85 ± 0.05	0.0007	1.56 (0.98–2.58)	1.99 (1.07–3.70) *	-
	Beta + TPP	819	0.55 ± 0.09	0.0028	1.79 (0.84–3.80)	1.76 (0.76–4.12)	-
Beta-R	Beta	554	1.27 ± 0.12	0.0097	13,146.98 (9406.70–18,374.45)	-	-
	Beta + PBO	539	2.21 ± 0.11	0.0023	1838.03 (1689.32–1997.67)	7.16 (5.07–10.11) *	2.82
	Beta + DEM	523	1.44 ± 0.16	0.0127	3944.69 (3268.18–4761.24)	3.33 (2.27–84.89) *	1.67
	Beta + TPP	573	1.78 ± 0.22	0.0143	5520.51 (4332.95–7033.55)	2.38 (1.58–3.60) *	-

Beta, beta-cypermethrin; *N*, Number of aphids tested; *p* Values, *p* < 0.05 indicates a significant linear relationship between the logarithm of insecticide dose and the probability of death. 95% CI, 95% confidence interval; SR, synergism ratio, SR = LC_50_ (insecticide alone)/LC_50_ (insecticide + synergist); *, the non-inclusion of 1 in the 95% CI indicated that the synergism ratio was significantly different; RSR, relative synergism ratio, RSR= SR of the Beta-R strain with the synergist/SR of the SS strain with the synergist.

**Table 5:** Toxicity determination parameters of beta-cypermethrin against wild-type (A-Dmel) and M918L mutant (T-Dmel) *Drosophila melanogaster*.

Strain	*N*	Slope ± SE	LC_50_ (mg/L) (95% CIs)	*p*-Values	RLR (95% CI)
T-Dmel	425	3.24 ± 0.39	5.20 (3.06–8.84)	0.0035	
A-Dmel	436	2.32 ± 0.30	1.75 (0.94–3.28)	0.0043	2.97 (1.30–6.74) *

***N***, Number of *D. melanogaster* tested; 95% CI, 95% confidence interval; *p*-Values, *p* < 0.05 indicates a significant linear relationship between the logarithm of insecticide dose and the probability of death. relative resistance ratio (RLR), RLR = LC_50_ of the T-Dmel/LC_50_ of the A-Dmel; *, the non-inclusion of 1 in the 95% CI indicates a significant difference in LC_50_ values of the insecticides between the two strains.

## Data Availability

The original data presented in this study are included in the article. Further inquiries can be directed to the corresponding author.

## Supplementary Materials

The following supporting information can be downloaded at: https://www.mdpi.com/article/10.3390/insects17101029/s1, Table S1: Primers used for the qRT-PCR analysis and for detecting mutations in the voltage-gated sodium channel gene. Table S2: The raw data on resistance levels to beta-cypermethrin for SS and Beta-R strains. Table S3: Raw data on the multiple resistance spectrum of SS and Beta-R strains of *R. padi*. Table S4: Raw data on the synergistic effects of TPP, DEM, and PBO in SS strains of *R. padi*. Table S5: Raw data on the synergistic effects of TPP, DEM, and PBO in Beta-R strains of *R. padi*. Table S6: Raw data on Toxicity determination parameters of beta-cypermethrin against A-Dmel and T-Dmel *D. melanogaster.* Text S1: Insecticides, Chemicals, and Synergists. Text S2: Life-table Analysis. Text S3: Enzyme Activity Analysis. Text S4: Detection of Mutations in the *R. padi* VGSC Gene. Text S5: Raw data on life table of SS strains. Text S6: Raw data on life table of Beta-R strains. Figure S1: Agarose electrophoretogram of PCR products of the RpVGSC-H1 and RpVGSC-H2 genes. Figure S2: Alignment of the predicted amino acid sequences encoded by the voltage-gated sodium channel gene H1 and H2 subunits examined in this study with the *R. padi* QTH36189.1 and QTH36190.1 sequence from the NCBI database. Figure S3: Alignment of sequences encoded by the voltage-gated sodium channel gene IIS4–S6 region in wild-type (A-Dmel) and M918 mutant (T-Dmel) *Drosophila melanogaster*.

## References

[1] Deng D., Duan W., Wang H., Zhang K., Guo J., Yuan L., Wang L., Wu S. (2019). Assessment of the effects of lethal and sublethal exposure to dinotefuran on the wheat aphid *Rhopalosiphum padi* (Linnaeus). Ecotoxicology.

[2] Wang K., Peng X., Zuo Y., Li Y., Chen M. (2016). Molecular cloning, expression pattern and polymorphisms of NADPH-cytochrome P450 reductase in the bird cherry-oat aphid *Rhopalosiphum padi* (L.). PLoS ONE.

[3] Bettaibi K., Mezghani-Khemakhem M., Bouktila D., Charaabi K., Raboudi F., Makni H., Makni M. (2016). A novel method for molecular targeting of insecticide resistance in *Rhopalosiphum padi* L. (Homoptera: Aphididae). Int. J. Pest Manag..

[4] Gong P., Li X., Gao H., Wang C., Li M., Zhang Y., Li X., Liu E., Zhu X. (2021). Field evolved resistance to pyrethroids, neonicotinoids, organophosphates and macrolides in *Rhopalosiphum padi* (Linnaeus) and *Sitobion avenae* (Fabricius) from China. Chemosphere.

[5] Chen X., Tie M., Chen A., Ma K., Li F., Liang P., Liu Y., Song D., Gao X. (2017). Pyrethroid resistance associated with M918 L mutation and detoxifying metabolism in *Aphis gossypii* from Bt cotton growing regions of China. Pest Manag. Sci..

[6] Fontaine S., Caddoux L., Brazier C., Bertho C., Bertolla P., Micoud A., Roy L. (2011). Uncommon associations in target resistance among French populations of *Myzus persicae* from oilseed rape crops. Pest Manag. Sci..

[7] Roush R.T., Mckenzie J.A. (1987). Ecological genetics of insecticide and acaricide resistance. Annu. Rev. Entomol..

[8] Zhang L., Shi J., Gao X. (2008). Inheritance of beta-cypermethrin resistance in the housefly *Musca domestica* (Diptera: Muscidae). Pest Manag. Sci..

[9] Sayyed A.H., Attique M.N.R., Khaliq A., Wright D.J. (2005). Inheritance of resistance and cross-resistance to deltamethrin in *Plutella xylostella* (Lepidoptera: Plutellidae) from Pakistan. Pest Manag. Sci..

[10] Saeed R., Abbas N., Hafez A.M. (2021). Biological fitness costs in emamectin benzoate-resistant strains of *Dysdercus koenigii*. Entomol. Gen..

[11] Gul H., Ullah F., Biondi A., Desneux N., Qian D., Gao X., Song D. (2019). Resistance against clothianidin and associated fitness costs in the chive maggot, *Bradysia odoriphaga*. Entomol. Gen..

[12] Wang K., Zhao J.N., Bai J.Y., Shang Y.Z., Zhang S.Q., Hou Y.F., Chen M.H., Han Z.J. (2021). Pyrethroid Resistance and Fitness Cost Conferred by the *super-kdr* Mutation M918L in *Rhopalosiphum padi* (Hemiptera: Aphididae). J. Econ. Entomol..

[13] Ffrench-Constant R.H., Bass C. (2017). Does resistance really carry a fitness cost?. Curr. Opin. Insect Sci..

[14] Amaral Rocha E.A., Silva R.M., Rodrigues da Silva B.K., Cruz C.G., Fernandes F.L. (2022). Fitness cost and reversion of resistance *Leucoptera coffeella* (Lepidoptera: Lyonetiidae) to chlorpyrifos. Ecotoxicol. Environ. Saf..

[15] Zhu X., Yang Y., Wu Q., Wang S., Xie W., Guo Z., Kang S., Xia J., Zhang Y. (2016). Lack of fitness costs and inheritance of resistance to *Bacillus thuringiensis* Cry1Ac toxin in a near-isogenic strain of *Plutella xylostella* (Lepidoptera: Plutellidae). Pest Manag. Sci..

[16] Wang K., Bai J., Zhao J., Su S., Liu L., Han Z., Chen M. (2020). Super-*kdr* mutation M918L and multiple cytochrome P450s associated with the resistance of *Rhopalosiphum padi* to pyrethroid. Pest Manag. Sci..

[17] Liu L., Wang S., Zuo J., Zhang X., Peng X., Wang K., Chen M., Anderson T. (2023). Characterization and fitness cost of bifenthrin resistance in *Rhopalosiphum padi* (Hemiptera: Aphididae). J. Econ. Entomol..

[18] Wei X., Pan Y., Xin X., Zheng C., Gao X., Xi J., Shang Q. (2017). Cross-resistance pattern and basis of resistance in a thiamethoxam-resistant strain of *Aphis gossypii* Glover. Pestic. Biochem. Physiol..

[19] Haddi K., Valbon W.R., Jumbo L.O.V., Oliveira L.O.D., Guedes R.N.C., Oliveira E.E. (2018). Diversity and convergence of mechanisms involved in pyrethroid resistance in the stored grain weevils, *Sitophilus* spp.. Sci. Rep..

[20] Panini M., Anaclerio M., Puggioni V., Stagnati L., Nauen R., Mazzoni E. (2015). Presence and impact of allelic variations of two alternative *s*-*kdr* mutations, M918T and M918L, in the voltage-gated sodium channel of the green peach aphid *Myzus persicae*. Pest Manag. Sci..

[21] Harrop T.W., Denecke S., Yang Y.T., Chan J., Daborn P.J., Perry T., Batterham P. (2018). Evidence for activation of nitenpyram by a mitochondrial cytochrome P450 in *Drosophila melanogaster*. Pest Manag. Sci..

[22] Ono M., Swanson J.J., Field L.M., Devonshire A.L., Siegfried B.D. (1999). Amplification and methylation of an esterase gene associated with insecticide-resistance in greenbugs, *Schizaphis graminum* (Rondani) (Homoptera: Aphididae). Insect Biochem. Mol. Biol..

[23] Haddi K., Tome H.V.V., Du Y., Valbon W.R., Nomura Y., Martins G.F., Dong K., Oliveira E.E. (2017). Detection of a new pyrethroid resistance mutation (V410L) in the sodium channel of *Aedes aegypti*: A potential challenge for mosquito control. Sci. Rep..

[24] Dong K., Du Y., Rinkevich F., Nomura Y., Xu P., Wang L., Silver K., Zhorov B.S. (2014). Molecular biology of insect sodium channels and pyrethroid resistance. Insect Biochem. Mol. Biol..

[25] Haddi K., Berger M., Bielza P., Cifuentes D., Field L.M., Gorman K., Rapisarda C., Williamson M.S., Bass C. (2012). Identification of mutations associated with pyrethroid resistance in the voltage-gated sodium channel of the tomato leaf miner (*Tuta absoluta*). Insect Biochem. Mol. Biol..

[26] Zuo Y., Peng X., Wang K., Lin F., Li Y., Chen M. (2016). Expression patterns, mutation detection and RNA interference of *Rhopalosiphum padi* voltage-gated sodium channel genes. Sci. Rep..

[27] Tang Q.Y., Zhang C.X. (2013). Data Processing System (DPS) software with experimental design, statistical analysis and data mining developed for use in entomological research. Insect Sci..

[28] Chi H. (1988). Life-table analysis incorporating both sexes and variable development rates among individuals. Environ. Entomol..

[29] Chi H., Liu H. (1985). Two new methods for the study of insect population ecology. Bull. Inst. Zool. Acad. Sin..

[30] Chi H. (2025). TWOSEX-MSChart.zip. https://lifetablechi.com/software.

[31] Chi H., Su H.Y. (2006). Age-Stage, two-Sex life tables of *Aphidius gifuensis* (Ashmead) (Hymenoptera: Braconidae) and its host *Myzus persicae* (Sulzer) (Homoptera: Aphididae) with mathematical proof of the relationship between female fecundity and the net reproductive rate. Environ. Entomol..

[32] Wei M.F., Chi H., Guo Y.F., Li X.W., Zhao L.L., Ma R.Y. (2020). Demography of *Cacopsylla chinensis* (Hemiptera: Psyllidae) reared on four cultivars of *Pyrus bretschneideri* (Rosales: Rosaceae) and *P*. *communis* pears with estimations of confidence intervals of specific life table statistics. J. Econ. Entomol..

[33] Tuan S.J., Lee C.C., Chi H. (2014). Population and damage projection of *Spodoptera litura* (F.) on peanuts (*Arachis hypogaea* L.) under different conditions using the age-stage, two-sex life table. Pest Manag. Sci..

[34] Xu L., Wu M., Han Z. (2014). Biochemical and molecular characterisation and cross-resistance in field and laboratory chlorpyrifos-resistant strains of *Laodelphax striatellus* (Hemiptera: Delphacidae) from eastern China. Pest Manag. Sci..

[35] Cui L., Wang Q., Qi H., Wang Q., Yuan H., Rui C. (2018). Resistance selection of indoxacarb in *Helicoverpa armigera* (Hubner) (Lepidoptera: Noctuidae): Cross-resistance, biochemical mechanisms and associated fitness costs. Pest Manag. Sci..

[36] Tang Q.L., Ma K.S., Hou Y.M., Gao X.W. (2017). Monitoring insecticide resistance and diagnostics of resistance mechanisms in the green peach aphid, *Myzus persicae* (Sulzer) (Hemiptera: Aphididae) in China. Pestic. Biochem. Physiol..

[37] Habig W.H., Pabst M.J., Jakoby W.B. (1974). Glutathione S-transferases. The first enzymatic step in mercapturic acid formation. J. Chem. Ecol..

[38] Bradford M. (1976). A rapid and sensitive method for the quantitation of microgram quantities of protein utilizing the principle of protein-dye binding. Anal. Biochem..

[39] Wang R., Zhang Q., Zhou X., Zhang M., Yang Q., Su Q., Luo C. (2022). Characterization of Field-Evolved Resistance to Afidopyropen, a Novel Insecticidal Toxin Developed from Microbial Secondary Metabolites, in *Bemisia tabaci*. Toxins.

[40] Schechtman H., Souza M.O. (2015). Costly inheritance and the persistence of insecticide resistance in *Aedes aegypti* populations. PLoS ONE.

[41] Wang K., Zhang M., Huang Y., Yang Z., Su S., Chen M. (2018). Characterisation of imidacloprid resistance in the bird cherry-oat aphid, *Rhopalosiphum padi*, a serious pest on wheat crops. Pest Manag. Sci..

[42] Stará J., Kocourek F. (2018). Seven-year monitoring of pyrethroid resistance in the pollen beetle (*Brassicogethes aeneus* F.) during implementation of insect resistance management. Pest Manag. Sci..

[43] Shang Q., Pan Y., Fang K., Xi J., Brennan J.A. (2012). Biochemical characterization of acetylcholinesterase, cytochrome P450 and cross-resistance in an omethoate-resistant strain of *Aphis gossypii* Glover. Crop Prot..

[44] Bi R., Pan Y., Shang Q., Peng T., Yang S., Wang S., Xin X., Liu Y., Xi J. (2016). Comparative proteomic analysis in *Aphis glycines* Mutsumura under lambda-cyhalothrin insecticide stress. Comp. Bioche Physiol. Part D.

[45] Mazari A.M.A., Mannervik B. (2016). Drosophila GSTs display outstanding catalytic efficiencies with the environmental pollutants 2,4,6-trinitrotoluene and 2,4-dinitrotoluene. Biochem Bio Rep..

[46] Sun H., Kasai S., Scott J.G. (2017). Two novel house fly Vssc mutations, D600N and T929I, give rise to new insecticide resistance alleles. Pestic. Biochem. Physiol..

[47] Karatolos N., Gorman K., Williamson M.S., Denholm I. (2012). Mutations in the sodium channel associated with pyrethroid resistance in the greenhouse whitefly, *Trialeurodes vaporariorum*. Pest Manag. Sci..

