# Supplementary material for: Characterization of a Stably Maintained, Highly Beta-Cypermethrin-Resistant Strain of the Bird Cherry-Oat Aphid Rhopalosiphum padi (Hemiptera: Aphididae)
Source: Insects. 2026 Oct 7;17(10):1029. doi: 10.3390/insects17101029 (PMC13705059; doi:10.3390/insects17101029)
Supplement: Supplementary file 1 [file insects-17-01029-s001.zip › Supplementary Materials.pdf]

Characterization of field-evolved, stably heritable beta-cypermethrin resistance in the bird cherry-oat aphid *Rhopalosiphum padi* (Hemiptera Aphididae)

Xinan Li [1, 2#], Peipan Gong [3#], Zilan Zhao [2 #], Fengfan Wang [1, 2], Qiuchi Li [2], Xiangrui Li [2], Yunhui Zhang [2*], Xun Zhu [4 *]

[1] Henan Engineering Technology Research Center for Major Invasive Alien Species Prevention and Control, School of Plant Protection and Environment, Henan Institute of Science and Technology, Xinxiang 453003, China.

[2] State Key Laboratory for Biology of Plant Diseases and Insect Pests, Institute of Plant Protection, Chinese Academy of Agricultural Sciences, Beijing, 100193, PR China.

[3] The Ministry of Agriculture and Rural Affairs Key Laboratory of Integrated Pest Management of Tropical Crops, Environment and Plant Protection Institute, Chinese Academy of Tropical Agricultural Sciences, Haikou 571101, China.

[4] State Key Laboratory for Quality and Safety of Agro-Products, Institute of Quality Standards and Testing Technology for Agro-Products, Chinese Academy of Agricultural Sciences, Beijing 100081, China.

[#] These authors contributed equally to this work.

[*] **Corresponding Authors:**

Ph. D Xun Zhu, Email: zhuxun@caas.cn

Ph. D Yunhui Zhang, Email: yhzhang@ippcaas.cn

Table S1. Primers used for the qRT-PCR analysis and for detecting mutations in the voltage-gated sodium channel gene

Primer name	Sequence (5′–3′)
*Rp*VGSC-H1F	ATGTCCATTGCTGACACCGATTC
*Rp*VGSC-H1R	CTAGCATTTTAAACTATCCAGG
*Rp*VGSC-H2F	ATGAGTGTGTACAGTAGTGAGG
*Rp*VGSC-H2R	TTAGACGTCGGCGAGTCTTGAGCT

Table S2. The raw data on resistance levels to beta-cypermethrin for the beta-cypermethrin-susceptible (SS) and beta-cypermethrin-resistant (Beta-R) strains.

Strain	Generation	Dose (mg/L)	mortality	Corrected mortality (%)
SS		0	0.08	
		1	0.38	33.11
		5	0.64	60.47
		25	0.78	76.22
		125	0.90	88.72
		625	0.98	97.99
Beta-R	G_0	0	0.02	
		5	0.05	3.25
		25	0.09	7.31
		125	0.29	27.92
		625	0.63	61.94
		1250	0.78	77.22
	G_4	0	0.03	
		5	0.07	4.05
		25	0.08	6.29
		125	0.28	26.60
		625	0.37	35.44
		1250	0.64	63.76
	G_7	0	0.03	
		150	0.06	2.77
		300	0.08	6.57
		600	0.20	18.37
		1200	0.52	51.51
		2400	0.76	75.63
	G_{10}	0	0.07	
		50	0.08	1.03
		200	0.15	8.36
		800	0.24	18.76
		3200	0.45	41.24
	G_{22}	0	0.02	
		1000	0.07	5.38
		2000	0.13	11.52
		4000	0.21	19.25
		8000	0.39	37.64
	G_{62}	0	0.02	
		1000	0.10	8.27
		2000	0.17	15.10
		4000	0.24	22.62
		8000	0.43	41.69

Table S3. Raw data on the multiple resistance spectrum of beta-cypermethrin-susceptible (SS) and beta-cypermethrin-resistant (Beta-R) strains of *Rhopalosiphum padi* after 62 generations.

Insecticides	SS strains			Beta-R strains		
	Dose (mg/L)	mortality	Corrected mortality (%)	Dose (mg/L)	mortality	Corrected mortality (%)
Lambda-cyhalothrin	0	0.07		0	0.06	
	4	0.48	44.28	4	0.09	3.47
	20	0.77	75.03	20	0.10	4.53
	100	0.87	85.66	100	0.19	13.36
	500	0.96	95.67	500	0.35	30.40
	2500	0.99	99.29	2500	0.47	43.11
Bifenthrin	0	0.08		0	0.02	
	1	0.33	26.98	2	0.03	0.72
	5	0.54	49.68	10	0.07	5.16
	25	0.72	70.09	50	0.15	13.17
	125	0.86	84.65	250	0.27	25.97
	625	0.98	97.99	1250	0.44	43.11
Sulfoxaflor	0	0.18		0	0.05	
	1	0.33	17.70	2	0.10	5.46
	5	0.59	49.67	10	0.18	13.51
	25	0.75	70.09	50	0.33	29.41
	125	0.93	91.74	250	0.56	54.16
	625	0.97	95.93	1250	0.86	85.13
Thiamethoxam	0	0.08		0	0.06	
	1	0.36	30.58	2	0.24	19.26
	5	0.58	54.01	10	0.37	33.40
	25	0.71	68.78	50	0.51	47.70
	125	0.83	82.02	250	0.63	61.13
	625	0.96	95.27	1250	0.82	81.02

Continued Table S3. Raw data on the multiple resistance spectrum of beta-cypermethrin-susceptible (SS) and beta-cypermethrin-resistant (Beta-R) strains of *Rhopalosiphum padi* after 62 generations.

Insecticides	SS strains			Beta-R strains		
	Dose (mg/L)	mortality	Corrected mortality (%)	Dose (mg/L)	mortality	Corrected mortality (%)
Imidacloprid	0	0.08		0	0.06	
	1	0.31	24.63	2	0.14	8.81
	5	0.48	43.94	10	0.26	21.20
	25	0.57	52.83	50	0.53	50.13
	125	0.79	76.75	250	0.71	69.09
	625	0.91	90.70	1250	0.87	85.92
Abamectin	0	0.11		0	0.06	
	1	0.17	7.03	2	0.23	17.76
	5	0.40	32.31	10	0.41	37.56
	25	0.88	86.40	50	0.60	57.45
	125	0.98	97.46	250	0.83	81.68
				1250	0.92	91.88
Omethoate	0	0.06		0	0.06	
	2	0.08	2.09	2	0.09	3.18
	10	0.22	17.04	10	0.21	15.54
	50	0.34	30.19	50	0.38	33.51
	250	0.61	58.16	250	0.80	78.34
	1250	0.91	90.03	1250	0.97	96.92
Chlorpyrifos	0	0.12		0	0.06	
	0.8	0.22	10.89	1	0.44	39.97
	1.6	0.25	15.25	5	0.84	82.84
	3.2	0.41	32.49	25	0.99	98.50
	6.4	0.97	97.03	125	1.00	100.00
	12.8	0.99	99.19			

Table S4. Raw data on the synergistic effects of TPP, DEM, and PBO in beta-cypermethrin-susceptible (SS) strains of *Rhopalosiphum padi* after 62 generations.

beta-cypermethrin + synergist	Dose (mg/L)	mortality	Corrected mortality (%)
beta-cypermethrin	0	0.08	
	1	0.38	33.11
	5	0.64	60.47
	25	0.78	76.22
	125	0.90	88.72
	625	0.98	97.99
beta-cypermethrin + PBO	0	0.08	
	1	0.52	48.02
	5	0.73	70.74
	25	0.88	86.78
	125	0.97	97.25
beta-cypermethrin + DEM	0	0.08	
	1	0.50	45.32
	5	0.61	57.54
	25	0.72	69.67
	125	0.78	75.76
	625	0.83	81.88
beta-cypermethrin + TPP	0	0.08	
	1	0.51	46.49
	5	0.58	54.01
	25	0.80	78.57
	125	0.84	82.80
	625	0.93	92.34

Table S5. Raw data on the synergistic effects of TPP, DEM, and PBO in beta-cypermethrin-resistant (Beta-R) strains of *Rhopalosiphum padi* after 62 generations.

beta-cypermethrin + synergist	Dose (mg/L)	mortality	Corrected mortality (%)
beta-cypermethrin	0	0.02	
	1000	0.10	8.27
	2000	0.17	15.10
	4000	0.24	22.62
	8000	0.43	41.69
beta-cypermethrin + PBO	0	0.00	
	1000	0.30	29.51
	2000	0.52	51.85
	4000	0.75	75.25
	8000	0.93	92.86
beta-cypermethrin + DEM	0	0.00	
	1000	0.20	19.79
	2000	0.35	35.29
	4000	0.45	45.16
	8000	0.70	69.64
beta-cypermethrin + TPP	0	0.00	
	1000	0.08	7.87
	2000	0.24	23.68
	4000	0.45	45.24
	8000	0.57	57.04

Table S6. Raw data on Toxicity determination parameters of beta-cypermethrin against wild-type (A-Dmel) and M918L mutant (T-Dmel) *Drosophila melanogaster*

T-Dmel strains			A-Dmel strains		
Dose (mg/L)	mortality	Corrected mortality (%)	Dose (mg/L)	mortality	Corrected mortality (%)
0	0.00		0	0.00	
0.2	0.00	0.00	0.2	0.04	3.57
1	0.07	6.76	1	0.25	25.40
5	0.51	51.32	5	0.79	79.37
25	0.88	88.24	25	0.97	97.26
125	1.00	100.00	125	1.00	100.00

Text S1. Insecticides, Chemicals, and Synergists

The insecticides used in this study were technical grade compounds. Beta-cypermethrin (95%), lambda-cyhalothrin (96%), bifenthrin (97%), imidacloprid (96%), thiamethoxam (97%), abamectin (95%), and chlorpyrifos (97%) were provided by Beijing Green Agricultural Science and Technology Group Co., Ltd. (China). Omethoate (40%, emulsifiable concentrate, EC) was provided by Hebei Xinxing Chemical Co., Ltd. (China). Sulfoxaflor (96%) was supplied by Hubei Kangbaotai Fine-Chemicals Co., Ltd. (China). The following additional materials used in this study were purchased from Sigma-Aldrich Shanghai Trading Co., Ltd. (China): N,N-dimethylformamide (DMF), piperonyl butoxide (PBO; reagent grade), triphenyl phosphate (TPP; reagent grade), diethyl maleate (DEM; reagent grade), α-naphthol, fast blue B salt, α-naphthyl acetate (α-NA), eserine, reduced glutathione (GSH), Coomassie brilliant blue G250, bovine serum albumin (BSA), 1-chloro-2,4-dinitrobenzene (CDNB), phenylmethylsulfonyl fluoride (PMSF), n-phenylthiourea (PTU), ethylenediaminetetraacetic acid (EDTA), and DL-dithiothreitol (DTT**).**

Text S2. Life-table Analysis

Life tables for the SS and Beta-R strains were established using the age-stage, two-sex life-table approach. For each strain, apterous adults that had not reproduced were placed on fresh wheat seedlings in a Petri dish (9 cm diameter) lined with a moistened filter paper for the reproductive period, with one adult per dish. After 24 h, all adult aphids were removed and a nymph was left in each dish for the life-table study. The molting and mortality of the aphids in the dishes were recorded every day. At maturity, the adult survival and fecundity (offspring/individual) were recorded. The newborn nymphs were counted and removed daily until the adult aphids died. The wheat seedlings were replaced by fresh seedlings every 3 days. The life-table bioassay was performed in a greenhouse at 20 ± 1 °C with 60 ± 10% relative humidity and a 16-h light:8-h dark photoperiod.

The raw life-table data for the *R. padi* Beta-R and SS strains were analyzed according to the age-stage, two-sex life-table theory using the TWOSEX-MSChart program. The analyzed data included the developmental durations of diverse life stages, longevity, fecundity, age-specific survival rate (l_x), intrinsic rate of increase (r), net reproductive rate (R_0), and mean generation time (T). The variance and standard error of the developmental durations, fecundity, and population parameters (r, R_0, and T) were calculated using the bootstrap procedure included in TWOSEX-MSChart, with 100,000 random resamplings. The differences between the Beta-R and SS strains were using the paired bootstrap test based on the CIs of the differences at the 5% significance level implemented in TWOSEX-MSChart. The relative fitness (r_f) of the Beta-R strain was calculated using the following equation: r_f=r of the Beta-R strain/r of the SS strain.

Text S3. Enzyme Activity Analysis

The carboxylesterase activity was determined using α-NA as previously described. The 250-μL reaction volume (per well) in a 96-well microplate contained 150 μL reaction mix (107 mM α-NA, 107 mM eserine, and 0.01 g mL^{-1} fast blue B salt), 50 μL PBS, and 50 μL enzyme solution. The absorbance was measured at 600 nm and 30 °C for 5 continuous minutes using the kinetic model of the Flex Station 3 microplate reader (USA).

The GST activity in apterous adults was determined using CDNB substrate as previously described, with a minor modification. Briefly, the 300-μL reaction mixture consisted of 100 μL CDNB (1.2 mM) substrate solution, 100 μL GSH (6 mM), and 100 μL diluted enzyme solution. The absorbance was measured at 340 nm for 10 min, with a read interval of 26 s, using the FlexStation 3 microplate reader.

An insect function oxidase ELISA kit (Huabaitai Biotechnology Corporation, Beijing, China) was used to assay the P450 levels as previously described, with a minor modification. The enzyme solution was transferred to a Microlon ELISA plate according to the manufacturer's instructions. The optical density (OD) was measured at 450 nm, and the P450 concentration was calculated based on a standard curve ($y = 0.72 + 0.0427x$) and then expressed as IU/L. The absorbance was measured using the FlexStation 3 microplate reader.

The protein content of the enzyme solution was determined using the Bradford method, with BSA as the standard. Briefly, 10 μL diluted solution for the three enzymes and 200 μL protein assay dye reagent was mixed. After 5 min, the OD at 595 nm was recorded using the FlexStation 3 microplate reader.

Text S4. Detection of Mutations in the *R. padi VGSC* Gene

Total RNA was isolated from 10 apterous adult aphids using the RNAprep pure Tissue Kit (Tiangen, Beijing, China), with five replicates per strain. Sample RNA concentrations were measured (at 260 nm) using the Nano Photometer N50 Touch (IMPLEN, München, Germany). Next, 1 mg RNA was used as the template to synthesize first-strand cDNA with the PrimeScript™ II 1st Strand cDNA Synthesis Kit (Takara, Dalian, China) following the manufacturer-recommended protocol.

The *R. padi VGSC* Gene was amplified from cDNA. Full-length *RpVGSC* sequences (*RpVGSC*-H1 and *RpVGSC*-H2) were amplified using gene-specific primer pairs (Table S1) and LA Taq Polymerase (Takara, Dalian, China). PCR was performed in a volume of 25 μL using standard procedures with 2 μL of each primer (10 mmol/L), 2.5 μL of 10× buffer (Mg^{2}+ Plus), 4 μL of dNTPs (0.2 mmol/L), 2 μL cDNA as the template, 0.25 μL of Takara LA-Taq DNA polymerase (LA Taq; Takara Bio, Dalian, China) (5 U/μL), and 12.25 μL of ddH_2O, which resulted in more efficient amplification and higher fidelity than conventional Taq DNA polymerase, under PCR conditions of 94 °C for 4 min, followed by 30 cycles of 94 °C for 30 s, 60 °C for 30 s, and 72 °C for 4 min, and a final 10 min at 72 °C. The PCR products were visualized by 1.0% agarose gel electrophoresis, and were purified using a Gel Extraction Kit (CWBIO, Jiangsu, China).

The purified fragments were then cloned into the pTOPO-TA Simple Cloning Kit (ThermoFisher, Beijing, China) and transformed into DH5*α* Competent Cell (CWBIO, Jiangsu, China). Finally, 10 positive clones were randomly selected from each sample, and the universal primer M13 was used for bidirectional sequencing analysis by Tsingke Biotech (Beijing, China). The sequencing results were used to determine the encoded amino acid sequences, which were then used to screen the NCBI database. Additionally, chromatographic peaks were checked using the Chromas software.

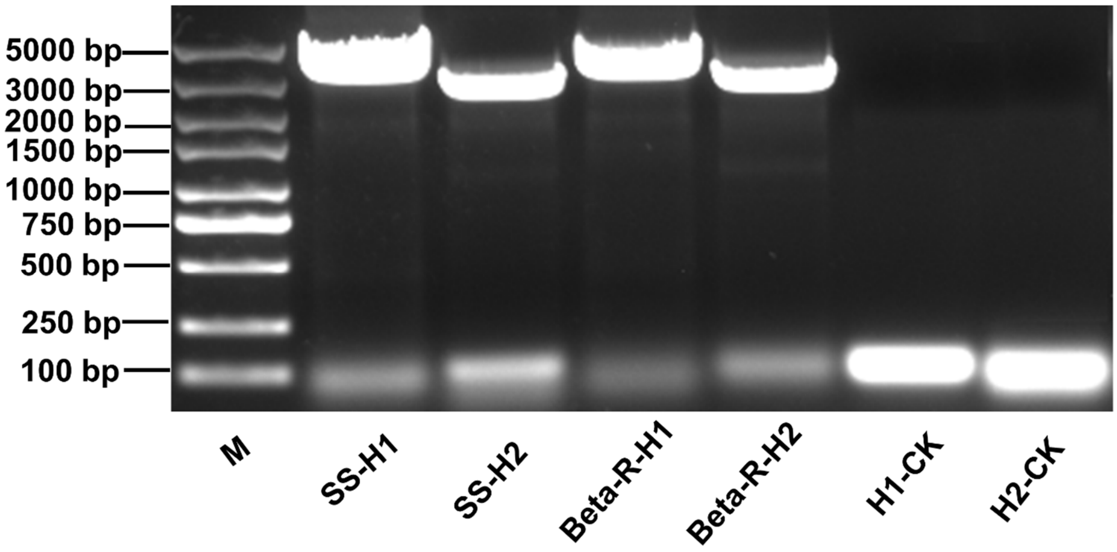

Figure S1. Agarose electrophoretogram of PCR products of the *RpVGSC*-H1 and *RpVGSC*-H2 genes.

```
QTH36189.1: MSIADTDSSFSSEEKSLFRPFTRESLRAIEQRIAEDHAKQKELEEKRAEGEVRYEDEDEDEGPQPDATLEQGAPLPVRLVGTFPPELASVPLEDIDPYYH
SS-H1:                ------------------------------------------------------------------------------------------
RR-H1:                ------------------------------------------------------------------------------------------
                                                Ⅰ S1                          Ⅰ S2
QTH36189.1: NQKTFVVISKGKDIFRFSATDGLWALDPFNPIRRVAIYILVHPIFSVTIITTILTNCVFMIMPPTPTIEASEVIFTGIYTFESAVKVMARGFILEHFTYL
SS-H1:      ----------------------------------------------------------------------------------------------------
RR-H1:      ----------------------------------------------------------------------------------------------------
               Ⅰ S3                Ⅰ S4                              Ⅰ S5
QTH36189.1: RDAWNWLDFIVIALAYVTMGIELGNLAVLRTFRVLRALKTVAIVPGLKTIVGAVIESVKNLRDVIILTIFSLSVFALLGLQIYMGVLTQKCIKYFPTDGS
SS-H1:      ----------------------------------------------------------------------------------------------------
RR-H1:      ----------------------------------------------------------------------------------------------------
                                                                      Ⅰ P-loop                   Ⅰ S6
QTH36189.1: AGNLTNENWFAFMSNSSNWQPGEEEPDEYPLCGNGTGAGQCKEGYMCIQGFGINPNYGYTSFDTFAWALLSAFRLMTQDNWEALYQQVLRAAGPWHMFFF
SS-H1:      ----------------------------------------------------------------------------------------------------
RR-H1:      ----------------------------------------------------------------------------------------------------

QTH36189.1: IVIIFLGSFYLVNLILAIVAMSYDELQKKAEEEEAAEEEAIREAEQAAKDREVRRQAHEERVAERAERARHVTQHPKSPSDFSCQSYDNMFADGQDRGIG
SS-H1:      ----------------------------------------------------------------------------------------------------
RR-H1:      ----------------------------------------------------------------------------------------------------

QTH36189.1: NDHHREKMSLRSVSITSHDKNSDTGSVDRQSGKTRKASLSLPGSPFNIRRASRGSHQLSHRNGRPRFTGADTKPLVLNTLLDAEEHLPYADDSNAVTPMS
SS-H1:      ----------------------------------------------------------------------------------------------------
RR-H1:      ----------------------------------------------------------------------------------------------------

QTH36189.1: EENGAIIVPVSYANFGSRHSSYTSHTSRITYTSHADLFKPPMTKERQLRSRSARNYFNPSDQRYHRDDDYDSSSMSKSKQKVDECGYNDSQKHTVVDMRD
SS-H1:      ----------------------------------------------------------------------------------------------------
RR-H1:      ----------------------------------------------------------------------------------------------------
                                                                                               Ⅱ S1
QTH36189.1: VMVLNDIIEQAAGRQSRGSEKAVSTVYVFPTDEDAVDGEDEEEEDEEPTFREKFQVWLLKFIDTFCVWDCGWPWLKFQQGLAFIVFDPFVELYITLCIVV
SS-H1:      ----------------------------------------------------------------------------------------------------
RR-H1:      ----------------------------------------------------------------------------------------------------
                              Ⅱ S2                            Ⅱ S3                    Ⅱ S4
QTH36189.1: NTLFMALDHHEMDPKLDFILNKANVFFSATFGVEAALKLMAMSPKYYFQMGWNIFDFIIVILSVVELLSAGYQGLSVLRSFRLLRVFKLAKSWPTLNLLI
SS-H1:      ----------------------------------------------------------------------------------------------------
RR-H1:      ----------------------------------------------------------------------------------------------------
                   Ⅱ S5                                              Ⅱ P-loop                        Ⅱ S6
QTH36189.1: SIMGRTIGALGNLTFVLCIIIFIFAVMGMQLFGKNYTEKMYLFKDHELPRWNFTDFLHSFMIVFRVLCGEWIESMWDCLHVGEPTCIPFFLATVVIGNLV
SS-H1:      --M-------------------------------------------------------------------------------------------------
RR-H1:      --L-------------------------------------------------------------------------------------------------

QTH36189.1: VLNLFLALLLSNFGSSNLSVPTADSDTNKITEAFERIGRFNKWMKTHIMNFLKALRAKITNQISVQASGRDRDIDLPVDETIVDVIAPFKDTKEPVEMTI
SS-H1:      ----------------------------------------------------------------------------------------------------
RR-H1:      ----------------------------------------------------------------------------------------------------

QTH36189.1: GDGMEFTIPGDVKQKIKKNQVGNSIGNHQGNKVGNDYKKESFDLDSLKC
SS-H1:      ---------------------------
RR-H1:      ---------------------------

QTH36190.1: MSVYSSEELLDAGIIYRNKKEQLDVTIGDGMELLIRGEKNKKKKPPTSSSYNSFGMHQSSTDENYYLKDKYEYDTRSTKSYGSHEHDPYDSESHRGSKRS
SS-H2:                ------------------------------------------------------------------------------------------
RR-H2:                ------------------------------------------------------------------------------------------
                                                                                                     ⅢS1
QTH36190.1: LHNAEEKKDPSKEDVEINQNILGGNDGIAAEEINGDEYKEQGPVEMVEDVFEEEEYPEDCFPPNCYKKFPPLAGDDETPFWLGWGQLRLKTFQLIENKYF
SS-H2:      ----------------------------------------------------------------------------------------------------
RR-H2:      ----------------------------------------------------------------------------------------------------
                                           ⅢS2                             ⅢS3                        ⅢS4
QTH36190.1: ETAVITMILLSSLALALEDVHLQHRPVLQDILYYMDRIFTVIFFLEMLIKWLALGFRNYFTNAWCWLDFIIVMVSLINFVAALLGASGIQAFKTMRTLRA
SS-H2:      ----------------------------------------------------------------------------------------------------
RR-H2:      ----------------------------------------------------------------------------------------------------
                                   ⅢS5                                                               ⅢP-loop
QTH36190.1: LRPLRAMARMQGMRVVVNALVQAIPSIFNVLLVCLIFWLIFAIMGVQLFAGKYYKCVDPKDGKTLNHEIIPDKNVCIAEMYKWENSKMNFDHVGNAYLCL
SS-H2:      ----------------------------------------------------------------------------------------------------
RR-H2:      ----------------------------------------------------------------------------------------------------
                                             ⅢS6
QTH36190.1: FQVATFNGWMEIMRDAVDSRDTHGKQPIREINNYMYFYFVFFIIFGSFFTLNLFIGVIIDNFNEQKKKTGASLEMFMTEDQKKYYNAMKKMSSKKPLKAI
SS-H2:      ----------------------------------------------------------------------------------------------------
RR-H2:      ----------------------------------------------------------------------------------------------------
                         ⅣS1                                 ⅣS2                             ⅣS3
QTH36190.1: PRPRWRPQSIVFQIVTDKKFDMLIMLFIGFNMLTMTLDHYQQTKLFTDVLERLNQIFIAIFSTECLLKIFALRYYYFKEPWNLFDFVVVILSLAGLVLSD
SS-H2:      ----------------------------------------------------------------------------------------------------
RR-H2:      ----------------------------------------------------------------------------------------------------
                     ⅣS4                                  ⅣS5                                       ⅣP-loop
QTH36190.1: LISKYFVSPTLLRVVRVAKVGRVLRLVKGAKGIRTLLFALAMSLPALFNICLLLFLVMFIFAIFGMSFFMNVDSHGGLDEDYNFRTFGQSMILLFMLSTS
SS-H2:      ----------------------------------------------------------------------------------------------------
RR-H2:      ----------------------------------------------------------------------------------------------------
                                            ⅣS6
QTH36190.1: SGWDAVLDGITNEENCQKPNLEMGITGSCGSSAVGTAFLLSYLVINFLIVINMYIAVILENYSQATEDVQEGLTDDDYDMYYEIWQHFDPNGTRYIRYDQ
SS-H2:      ----------------------------------------------------------------------------------------------------
RR-H2:      ----------------------------------------------------------------------------------------------------

QTH36190.1: LSDFLDILEPPLKIHKPNKYKIVSMDIPICKGDLIYCVDILDALTKDFFARKGNPIIETVAEIGEMQTRPEEAGYEPISSSLWRMREVYCAIIIQNSWRK
SS-H2:      ----------------------------------------------------------------------------------------------------
RR-H2:      ----------------------------------------------------------------------------------------------------

QTH36190.1: YTAAAKQQTADDERSDGAASPDGRETAVLVESDGFVTKNGHKVVIHSRSSSKSSRLADV
SS-H2:      -----------------------------------------------
RR-H2:      -----------------------------------------------
```

Figure S2. Alignment of the predicted amino acid sequences encoded by the voltage-gated sodium channel gene H1 and H2 subunits examined in this study with the *R. padi* QTH36189.1 and QTH36190.1 sequence from the NCBI database.

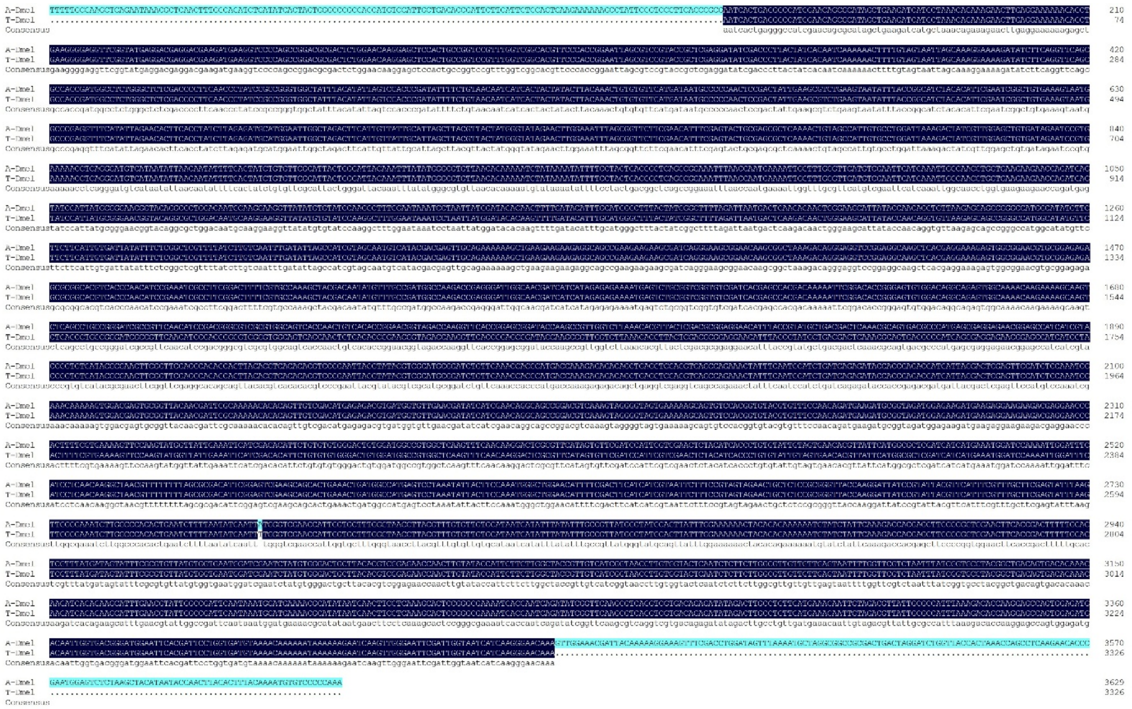

Figure S3. Alignment of sequences encoded by the voltage-gated sodium channel gene IIS4–S6 region in wild-type (A-Dmel) and M918 mutant (T-Dmel) *Drosophila melanogaster*.
